# Power, data and social accountability: defining a community‐led monitoring model for strengthened health service delivery

**DOI:** 10.1002/jia2.26374

**Published:** 2024-10-24

**Authors:** Ndivhuwo Rambau, Soeurette Policar, Alana R. Sharp, Elise Lankiewicz, Allan Nsubuga, Luke Chimhanda, Anele Yawa, Kenneth Mwehonge, Donald Denis Tobaiwa, Gérald Marie Alfred, Matthew M. Kavanagh, Asia Russell, Solange Baptiste, Onesmus Mlewa Kalama, Rodelyn M. Marte, Naïké Ledan, Brian Honermann, Krista Lauer, Nadia Rafif, Susan Perez, Gang Sun, Anna Grimsrud, Laurel Sprague, Keith Mienies

**Affiliations:** ^1^ Treatment Action Campaign (TAC) Johannesburg South Africa; ^2^ L'Organisation de Développement et de Lutte contre la Pauvreté (ODELPA) Port‐au‐Prince Haiti; ^3^ O'Neill Institute for National and Global Health Law, Georgetown Law Center Georgetown Medical Center Washington DC USA; ^4^ Andelson Office of Public Policy amfAR Washington DC USA; ^5^ Sexual Minorities Uganda (SMUG) Kampala Uganda; ^6^ Zimbabwe PEPFAR‐funded CLM Harare Zimbabwe; ^7^ Coalition for Health Promotion and Social Development (HEPS) Kampala Uganda; ^8^ Jointed Hands Welfare Organisation (JHWO) Harare Zimbabwe; ^9^ Action Citoyenne pour l'Egalité Sociale en Haïti (ACESH) Marchand Dessalines Haiti; ^10^ School of Public Health, Georgetown University Washington DC USA; ^11^ Health Global Access Project (Health GAP) Washington, DC USA; ^12^ International Treatment Preparedness Coalition Global (ITPC) Johannesburg South Africa; ^13^ Eastern Africa National Networks of AIDS and Health Service Organisations (EANNASO) Arusha Tanzania; ^14^ Bangkok Thailand; ^15^ The Global Fund to Fight AIDS, Tuberculosis and Malaria Geneva Switzerland; ^16^ The Joint United Nations Programme on HIV/AIDS (UNAIDS) Geneva Switzerland; ^17^ The International AIDS Society Cape Town South Africa

**Keywords:** accountability mechanisms, community advocacy, community‐led monitoring, health service delivery, health systems, social accountability

## Abstract

**Introduction:**

Despite international commitment to achieving the end of HIV as a public health threat, progress is off‐track and existing gaps have been exacerbated by COVID‐19's collision with existing pandemics. Born out of models of political accountability and historical healthcare advocacy led by people living with HIV, community‐led monitoring (CLM) of health service delivery holds potential as a social accountability model to increase the accessibility and quality of health systems. However, the effectiveness of the CLM model in strengthening accountability and improving service delivery relies on its alignment with evidence‐based principles for social accountability mechanisms. We propose a set of unifying principles for CLM to support the impact on the quality and availability of health services.

**Discussion:**

Building on the social accountability literature, core CLM implementation principles are defined. CLM programmes include a community‐led and independent data collection effort, in which the data tools and methodology are designed by service users and communities most vulnerable to, and most impacted by, service quality. Data are collected routinely, with an emphasis on prioritizing and protecting respondents, and are then be used to conduct routine and community‐led advocacy, with the aim of increasing duty‐bearer accountability to service users. CLM efforts should represent a broad and collective community response, led independently by impacted communities, incorporating both data collection and advocacy, and should be understood as a long‐term approach to building meaningful engagement in systems‐wide improvements rather than discrete interventions.

**Conclusions:**

The CLM model is an important social accountability mechanism for improving the responsiveness of critical health services and systems to communities. By establishing a collective understanding of CLM principles, this model paves the way for improved proliferation of CLM with fidelity of implementation approaches to core principles, rigorous examinations of CLM implementation approaches, impact assessments and evaluations of CLM's influence on service quality improvement.

## 1 INTRODUCTION

Progress against infectious diseases like HIV, tuberculosis and malaria has been extraordinary yet uneven, with profound inequities remaining across geographies, age groups, genders, sexual orientations and socio‐economic groups [1]. Strategies to remove barriers to accessing and remaining in care are urgently needed to deliver quality prevention and treatment services, with dignity, for all. Community advocacy has long played a role in improving healthcare services, with activists using a variety of mechanisms to demand greater accountability from governments, donors and health systems [2, 3, 4].

**Figure 1:**
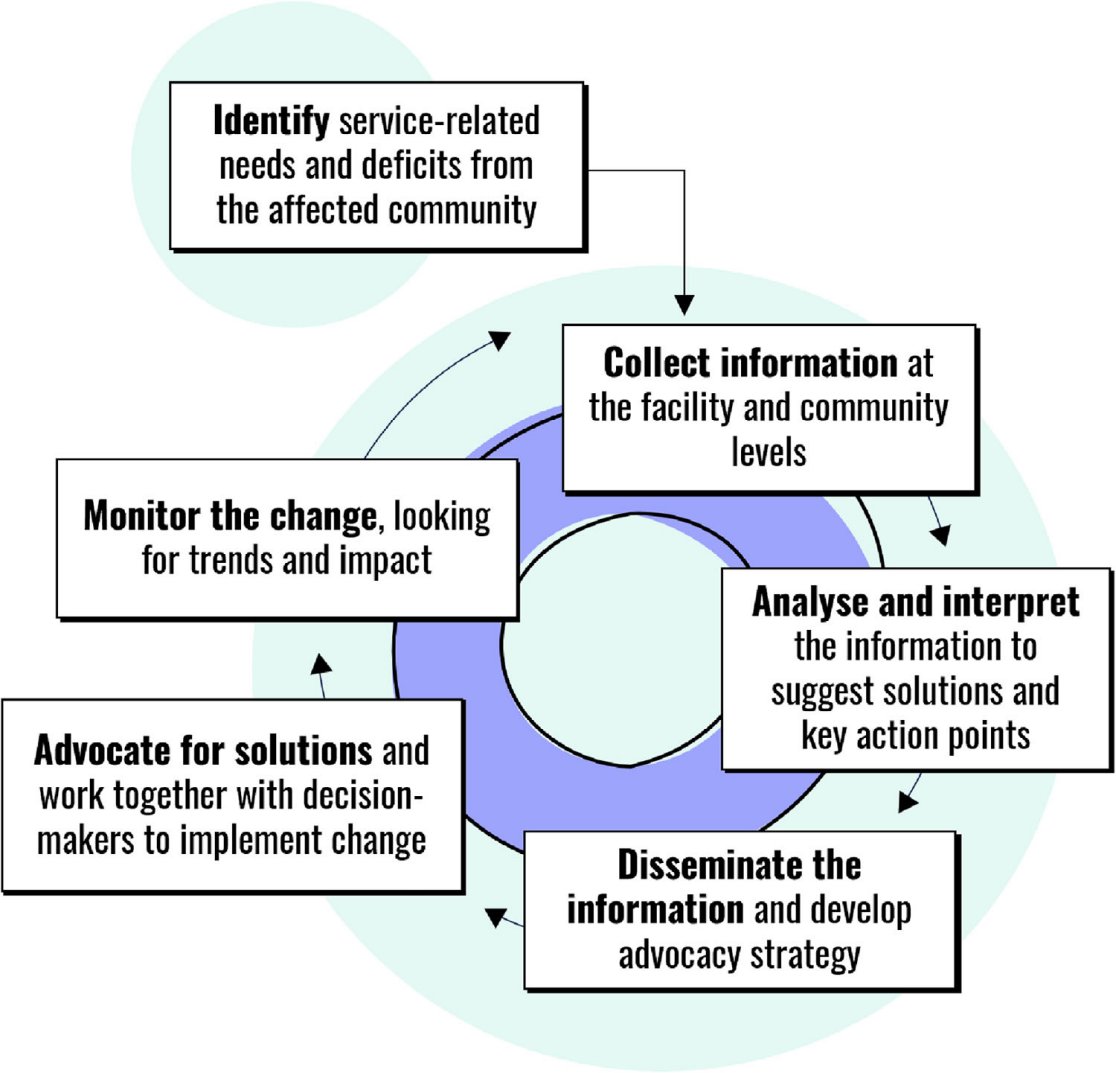
Phases of the CLM model. Integration of community‐led monitoring into service review and improvement [9].

Social accountability mechanisms are civil society‐led actions designed to hold governments accountable for effective service delivery, to improve governance and transparency, and to empower communities [5]. Several social accountability approaches have gained prominence in global health and international development [6, 7, 8], including participatory health committees, community scorecards, complaint mechanisms and community‐led monitoring (CLM) [5]. The CLM cycle involves local community‐led organizations (CLOs) and civil society leading a regular process of data collection, identifying issues, developing solutions, conducting advocacy and monitoring change to improve access and quality of services (Figure 1) [9, 10, 11, 12, 13, 14, 15].

In recent years, major global health donors, including PEPFAR and the Global Fund, have begun investing in CLM programmes [13, 14, 15]. However, as CLM programmes expand worldwide, the ability to critically compare implementation approaches and evaluate impact on health service delivery has been hindered by the challenges of comparing substantively different approaches, in diverse contexts, across different countries [7]. Indeed, the evidence on the impact of CLM in health systems is limited, with [16, 17, 18] most peer‐review publications focused on describing the implementation approaches used [19, 20, 21] or reporting the findings from CLM [19, 20, 22, 23]. While one study has reported on the implementation approaches considered best practices by CLM implementers [24], there remains a lack of evidence of when and how CLM impacts health systems.

Building on both the relevant social accountability literature and experiences implementing CLM for HIV, we propose a set of evidence‐informed principles for CLM implementation. These principles are intended to support impactful CLM implementation, while also facilitating future evaluations of CLM implementation and impact. The principles were thematically derived from the scientific literature on social accountability, grey literature from CLM programmes and guided by the authors’ experiences as CLM implementers, technical assistance providers, donors and other key partners. While the principles outlined in this document are drawn primarily from the HIV perspective, many are applicable to CLM in the health system more broadly.

## 2 DISCUSSION

### 2.1 CLM and social accountability

Historically, service users have often been excluded from pathways to directly engage with the duty‐bearers that fund and administer healthcare. Instead, healthcare users must lobby politicians, who may in turn influence civil servants and health services provision. This indirect pathway is termed the “long” route to accountability [6], and in most contexts, this distance between service user and duty‐bearer impedes communities’ ability to successfully advocate for improved service provision. In contexts with stigmatization, criminalization and human rights violations, health service users face even greater barriers to engaging in formal decision‐making spaces [25].

One tool for improving the quality of care is to create more direct routes of engagement by strengthening accountability through evidence‐informed advocacy [6, 26]. Accountability is the creation of a relationship between those with a duty of accountability (for instance, public officials and health service providers) and those individuals impacted by the duty‐bearer's actions [27]. Where communities lead these demands for accountability in an ongoing manner outside of formal political processes, the approach is termed “social accountability” [26, 28, 29].

Social accountability interventions act on health systems by improving “answerability” and “enforceability,” with the ultimate objective of rectifying power imbalances between institutions and civil society [7, 30, 31, 32, 33]. Answerability is a mandate imposed on duty‐bearers to describe and justify their actions, typically within multi‐stakeholder spaces [34]. Enforceability is the ability to use penalties, whether reputational, administrative or legal [35], to support the implementation of desired actions or remedies. CLM uses data and advocacy to drive duty‐bearers to respond to community needs and holds accountable those they have authority over.

### 2.2 Core principles of CLM implementation

Building on the social accountability literature and emerging lessons learned from CLM implementation, we propose five key CLM principles. These principles intentionally grant broad flexibility to communities in defining context‐appropriate implementation approaches, while defining guardrails to ensure the integrity of programme implementation.

1. CLM is led by funded, capacitated and directly impacted communities.
2. Community monitors are trained and supported service users.
3. Data are independently owned by communities and do not duplicate government and donor data unless for triangulation purposes.
4. CLM programmes must adhere to ethical data collection.
5. CLM includes advocacy activities aimed at generating accountability.

### 2.3 CLM is led by directly impacted communities who are fully funded and capacitated

A successful social accountability intervention is one that creates pathways for communities to directly advocate to duty‐bearers. The leadership of CLM programmes and agenda setting by directly impacted communities is, therefore, a core strategy for building direct pathways to duty‐bearers and ensuring that advocacy messages prioritize the community. Furthermore, community leadership makes CLM programmes more likely to achieve impact on health systems [36], in part due to duty‐bearers being more responsive to actors perceived to have “representative legitimacy,” with the strongest messengers being individuals who have expertise in the issues facing the healthcare system, knowledge of marginalized communities and who are perceived as representing a united front [37, 38, 39]. Regardless, the ongoing CLM cycle is often an operational challenge, and many efforts have been hindered by insufficient funding and capacity [7, 40]. As such, CLM programmes should be coordinated by an organized community‐owned civic structure capacitated to manage programmatic and financial components. Where partner institutions outside of the CLM programme are engaged in addressing capacity challenges (for instance, technical skills collecting and analysing data, administrative infrastructure, human resources), the focus of this support must be on building CLO capacity to lead these functions, rather than shifting ownership of aspects of the programme to non‐CLO partners. One narrow exception is where partner organizations act as a funding pass‐through on behalf of CLOs on a temporary basis, while CLOs build capacity to receive direct funding, or in criminalized contexts where CLOs cannot be legally recognized or organized. Additionally, insufficiently funding the full cycle of CLM activities has emerged as a key challenge that undermines the full potential of community data—especially leveraging insights for advocacy [40].

Notably, while duty‐bearers may seek to exert control over CLM narratives and methods, often to avoid public criticism, challenges to community leadership of programmes are often driven by misconceptions of the model. This is often due to stakeholders misunderstanding the fundamental role of community leadership in social accountability initiatives, due to perceiving CLM as monitoring and evaluation (M&E), a traditional service improvement intervention or research [24, 35, 41]. While CLM and M&E are both concerned with measuring facets of healthcare delivery, CLM emphasizes a community‐owned, participatory process of gathering information to advocate for greater accountability of health systems to communities. By contrast, M&E is traditionally understood as a measurement of the effectiveness and impact of programmes, generally at the behest of a government or donor. Donors and governments have a vested interest in the success of CLM, both as financial investors and as healthcare delivery organizations, and this support may be catalytic where it strengthens technical capacity and facilitates stakeholder engagement. However, ensuring community ownership is a core component of the model and is an important strategy for mitigating conflicts of interest [42].

### 2.4 CLM community monitors are trained and supported service users

As with programme leadership, community members themselves must be responsible for gathering data. This both reinforces community ownership and strengthens data collection, since peer interviewers may reduce self‐presentation bias and social desirability bias, particularly when interviews are conducted in private settings [43, 44]. Evidence from Uganda finds accountability impact is achieved when impacted populations themselves are responsible for both collecting and disseminating data [45].

In many programmes, data collectors are also the visible face of the programme, serving as the main point of contact with service providers; in South Africa, for instance, community monitors are responsible for data collection, solution generation and advocacy [46]. Accordingly, the data collectors’ proximity to healthcare users and communities is tied to the project's perceived legitimacy to duty‐bearers. Given their integral role in CLM, community monitors must be supported with continuous training and full and fair financial remuneration [40]. This capacity building is imperative for ensuring high‐quality data, reducing staff turnover, and may also increase service demand and health literacy among the monitors themselves and their community [40, 47].

### 2.5 Data are independently owned by communities and do not duplicate government and donor data unless for triangulation purposes

The exact types of data collected as part of CLM are not prescribed, and indeed, the data collection tools and sampling approaches are certain to vary by context; in practice, many CLM projects rely extensively on both quantitative and qualitative data collection [24, 40]. However, data collection for CLM must involve the active and meaningful engagement of service users in developing CLM frameworks, data collection tools and collecting information and must avoid undue influence from external actors [7, 36]. Decisions about how to share community data lie with the community and must maintain a level of aggregation that protects the confidentiality of respondents. Regardless of dissemination strategy, data collection systems must maintain independence from duty‐bearers, to avoid the risk and perception of conflict of interest, and decision‐making authority about how to publicize findings should remain with the CLO implementers [48].

While data collected by governments and international donors serve useful functions, these data are unlikely to be sufficient as an accountability tool for several reasons. First, granular data on health system functioning are often inaccessible in information‐poor contexts; second, information concerning vulnerable populations are unlikely to be adequately measured in stigmatized or criminalized contexts; and finally, government‐ and donor‐owned data systems designed to expose poor performance or corruption are themselves vulnerable to underperformance and conflicts of interest [48]. Additionally, existing M&E systems are typically not focused on identifying the actionable drivers of poor performance or quality of care and do not include data collection in community spaces outside of clinics. In general, unless there is an explicit community interest in verifying the accuracy of existing M&E, CLM should not duplicate government or donor data collection, but should instead gather targeted data that are clearly tied to proposed actions designed to address challenges [18].

Critically, for data to be useful, they must have three features: data must be relevant to service users, salient for duty‐bearers and sufficiently accessible to both groups that they may be acted on [18], and must specify the locations requiring intervention. Ensuring that data are linked directly to the localities that communities are working in is critical for providing duty‐bearers with actionable information on the locations that require intervention.

### 2.6 CLM programmes must adhere to ethical data collection

CLM data gathering must adhere to ethical practices of beneficence, respect for persons, justice and safety. This includes minimizing collection of personally identifiable information, limiting the burden on respondents (including patients, clinic staff and other duty‐bearers), ensuring informed consent, protecting community leadership in the analysis and interpretation of data, and sharing data transparently in a format that facilitates use [49].

In contexts where respondents may be placed at risk for their participation, such as in criminalized settings, extra care must be taken to protect the safety, security and individual confidentiality [18, 50]. While information such as users’ behavioural practices may be important for tailoring health systems programming, this does not mean that CLM is inherently the correct methodology by which to collect those data. In these contexts, it may be appropriate for CLM projects to partner with research programmes or local universities with additional ethical oversight.

### 2.7 CLM includes advocacy activities aimed at generating accountability

The existence of evidence alone is insufficient to create change [51]. After data have been collected and analysed, specific solutions and evidence‐based advocacy messages must be developed in response to challenges identified in data analysis [35]. Since duty‐bearers are more likely to implement recommendations that are locally relevant and defined by impacted populations, this process must be collaborative and inclusive [7, 38]. In practice, some CLM programmes hold workshops within community consortia to develop proposed solutions prior to engaging with duty‐bearers [52]. Advocacy strategies may also require multi‐level engagement; for instance, in a West African treatment observatory, CLM implementers conduct facility‐level advocacy as well as engagement with Global Fund Country Coordinating Mechanisms, USAID, networks of people living with HIV and national AIDS control programmes [53]. Similarly, in Zimbabwe, a National Steering Committee of duty‐bearers meets quarterly to discuss unresolved issues raised by the CLM programme [54].

While specific advocacy strategies vary, recommendations must be shared with duty‐bearers for collaborative problem‐solving. Since impactful change beyond micro‐level outcomes (e.g. facility‐level provider friendliness) often depends on prolonged and ongoing engagements, early and regular communication between CLM implementers and government officials has been described as key, with the data and recommendations seen as additive to existing government data sources (Evaluation of Ritshidze Community‐led Monitoring Programme in South Africa, unpublished). Many accountability efforts find improved receptivity when framed as a collaborative effort between service users and providers [7, 38, 55, 56].

The feasibility of collaborating with health system actors varies depending on the context [35]. For example, in politically resistant environments or when addressing issues of fraud, advocacy efforts may become more acrimonious [57]. Where duty‐bearers are not responsive to CLM, a second phase of creating “costs” for poor behaviours should follow, by imposing reputational costs and administrative or legal censure [35]. In practice, this may range from publicly identifying poor performers to public‐facing meetings, demonstrations and legal action [57]. Involvement with media has emerged as a useful tool in amplifying local concerns to a broader audience or citizen journalism approaches [56, 58, 59]. Of note, coalition building is a key driver of successful advocacy, usually involving affected populations, formal and informal community associations, and CLOs [7].

Several assumptions underlie the advocacy principle, notably that duty‐bearers are empowered and resourced to improve services and that service provision and policymaking are elastic in response to feedback [18]. Where there are no legal protections for free speech or legal frameworks to address professional misconduct and corruption, CLM programmes will likely have to rely more on the strengthening of relationships between providers and the community and expect that this expanded collaboration will take longer to result in service improvement, or rely on alternative non‐confrontational approaches to collective action [60].

## 3 CONCLUSIONS

A perennial issue in the social accountability literature is the challenge of comparing interventions and measuring impact, a process complicated by the wide diversity of efforts existing under the umbrella of community‐led accountability work [18]. This piece proposes a series of core principles for CLM implementation, with the aim of creating a shared understanding of the CLM model built on the social accountability literature. By creating a shared understanding of the principles of CLM, this framing will allow for future rigorous inquiry into comparative implementation approaches, assessments of impact, evaluations of the impact of CLM on health systems strengthening and the expansion of quality CLM implementation worldwide.

## COMPETING INTERESTS

SP and KM are employed by the funder of this publication, The Global Fund to Fight AIDS, Tuberculosis and Malaria. No other authors have competing interests to declare.

## AUTHORS’ CONTRIBUTIONS

AG: review and editing; AN: review and editing; AR: writing—original draft (supporting), review and editing; ARS: conceptualization, writing—original draft (lead); AY: review and editing; BH: writing—original draft (supporting), review and editing; EL: conceptualization, writing—original draft (lead); DDT: review and editing; GMA: review and editing; GS: review and editing; KL: review and editing; KM (Global Fund): conceptualization, writing—original draft (supporting), review and editing; KM (HEPS): review and editing; LC: review and editing; LS: review and editing; MMK: conceptualization, writing—original draft (supporting), review and editing; NL: review and editing; NR (TAC): writing—original draft (lead), review and editing; NR (ITPC): review and editing; SP (ODELPA): conceptualization, review and editing; SP (Global Fund): conceptualization, writing—original draft (supporting), review and editing; OMK: review and editing; RMM: review and editing; SB: review and editing; SP: review and editing.

## FUNDING

This publication was developed with support from The Global Fund to Fight AIDS, Tuberculosis and Malaria under the Community‐led Monitoring Strategic Initiative.

## Data Availability

No data were used in the production of this manuscript.
